# Outcome of Standardbred racehorses following femoropatellar arthroscopy for osteochondrosis dissecans

**DOI:** 10.1111/vsu.70058

**Published:** 2025-11-21

**Authors:** Adrienne D. Rhodes, Annette M. McCoy, Matthew C. Stewart, Santiago D. Gutierrez‐Nibeyro

**Affiliations:** ^1^ Department of Veterinary Clinical Medicine, College of Veterinary Medicine University of Illinois Urbana‐Champaign Illinois USA

## Abstract

**Objective:**

To evaluate postoperative racing performance of a population of Standardbred racehorses following arthroscopic removal of an osteochondrosis dissecans (OCD) lesion of the femoropatellar joint.

**Study design:**

Retrospective study.

**Sample population:**

A total of 45 client‐owned Standardbred racehorses.

**Methods:**

OCD lesions were measured and graded using the length of the subchondral bone defect from preoperative radiographs. Postoperative racing results were obtained from the United States Trotting Association, and follow‐up owner surveys were conducted. A control group of contemporaneous paternal siblings free of OCD lesions was selected for comparison of racing performance. Regression analysis was used to determine associations between presence or grade of OCD lesions and performance parameters with sex and gait covariates.

**Results:**

There was no significant difference in proportion of horses starting a race based on OCD lesion grade. Affected racehorses had fewer starts at 3 years of age (IRR = 0.84 [95% CI: 0.74–0.96], *p* = .012) but not at 2 years of age, when compared to unaffected siblings. There was also no difference in race wins or earnings between affected and unaffected horses.

**Conclusion:**

Arthroscopy remains an effective treatment for OCD lesions of the femoropatellar joint in Standardbred racehorses, when performed prior to the start of intensive training, and lesions treated in this manner have no major impact on racing potential. Limitations included a small number of affected individuals especially with higher grades of OCD lesions and there was no conservative treatment group.

**Clinical significance:**

OCD lesions within the femoropatellar joint in Standardbred racehorses resulted in minimal impact on postoperative racing performance when removed arthroscopically.

## 1 INTRODUCTION

Osteochondrosis dissecans (OCD) is a developmental orthopedic disease where there is osteochondral necrosis and separation of an osteochondral fragment from the parent bone following failure of endochondral ossification in the articular‐epiphyseal cartilage complex.^1^ OCD predominantly affects the tibiotarsal, the metacarpo‐(metatarso‐) phalangeal, and the femoropatellar joints of horses.^2^ Radiography and ultrasonography are clinically useful imaging modalities to identify most OCD lesions, including lesions within the femoropatellar joint.^3^ Standardbred and Thoroughbred racehorses are routinely screened via fluoroscopy and/or radiography to rule out the presence of OCD lesions before training. However, arthroscopic surgery is the gold standard technique for evaluation of the articular surfaces and definitive diagnosis of OCD lesions within a joint.^4^ Surgical debridement is the mainstay treatment of femoropatellar OCD lesions, although there are reports of conservative management.^2^, ^5^ Arthroscopic removal of osteochondral fragments has been the preferred treatment for OCD lesions of the femoropatellar joint since it was first described in 1985.^6^, ^7^ In very select cases, reattachment of cartilage flaps with absorbable pins can be performed.^8^, ^9^

Foland et al. reported lesion characteristics including size and location, and the postoperative outcome of a population of mostly Thoroughbred horses following arthroscopic removal of OCD lesions of the femoropatellar joint.^6^ In this study, the most common lesion location was the lateral trochlear ridge of the femur, and the lesions were assigned a grade (1–3) based on the radiographic length of the subchondral bone defect.^6^ Postoperatively, 64% of horses returned to or achieved their intended level of performance and horses with a grade 1 lesion had a significantly higher success rate (78%) compared to those with grade 2 or 3 lesions (63% and 54%, respectively).^6^ Clarke et al. also reported the postoperative outcome of Thoroughbred racehorses that underwent arthroscopic removal of femoropatellar OCD lesions and found that horses that were operated at <2 years of age had decreased performance including fewer starts, decreased earnings, and decreased wins at 3 years of age, compared to matched controls.^10^ A higher OCD lesion grade was associated with fewer wins at 3 years of age, but was not significantly associated with any of the other performance outcomes studied.^10^ Kerbert et al. also evaluated racing and sales performance of Thoroughbred racehorses following arthroscopic removal of OCD lesions of the lateral trochlear ridge of the femur as yearlings.^11^ Horses that went to a public sale after surgery had a significantly higher number of starts, wins, and earnings compared to those which did not go to the sale, as a significantly higher percentage of these horses had raced.^11^ Lesion size had no effect on the number of days from surgery until the first race, number of starts, wins and total earnings between the affected horses that went to public sale and the horses that did not. Affected horses and matched controls were sold for the same price and did not differ in their proportion that started racing at 2 years of age.

These previous studies have involved mostly Thoroughbred racehorses. The impact of femoropatellar OCD on the postoperative racing performance of Standardbred racehorses is less well documented and predominantly anecdotal. The objective of this study was to evaluate the postoperative racing performance of a population of Standardbred racehorses following arthroscopic removal of an OCD lesion of the femoropatellar joint. Additionally, we wanted to identify any potential prognostic indicators that would assist clinicians in their recommendation to clients. We hypothesized that horses with larger OCD lesions and involvement of multiple joints would have poorer racing performance (i.e., fewer starts, lower earnings, etc.).

## 2 MATERIALS AND METHODS

### 2.1 Study population

Medical records of Standardbred racehorses undergoing unilateral or bilateral femoropatellar arthroscopy for removal of OCD lesions at the University of Illinois Veterinary Teaching Hospital between January 2014 and January 2022 were searched and reviewed. The information obtained from the medical records included age, sex, joint(s) operated on, and diagnosis.

### 2.2 Radiographic measurements and grading

To determine OCD lesion severity, the length of the OCD lesions, defined as the subchondral bone defect, was independently measured on preoperative digital radiographs (lateromedial projections) by two observers (a board‐certified large animal surgeon and an equine surgery resident) working independently. The mean length of these two observations was used as the lesion measurement for data analysis. In a select number of cases, intraoperative radiographs were used for measurements when preoperative radiographs were unavailable. If a joint had more than one lesion present (i.e., lateral trochlear ridge and patella), only the lateral or medial trochlear ridge lesion was measured. Using the same grading scale developed by Foland et al. each lesion was assigned a grade (grade 1–3): grade 1 lesions were those <2 cm in length; grade 2 lesions were those ≥2 cm and <4 cm in length; and grade 3 lesions were those ≥4 cm in length.^6^ For horses with bilateral femoropatellar OCD lesions, the largest lesion size was used for analysis. Due to a small number of horses with grade 3 lesions, a modified grading scale was used in which grade 1 lesions remained lesions <2 cm in length but grade 2 lesions were lesions ≥2 cm.

### 2.3 Performance records

Postoperative racing results for Standardbred racehorses were obtained from the United States Trotting Association online database.^12^ Information obtained from racing records included date of birth (if different from medical records), gait, fastest recorded time and the number of race starts, race wins, and earnings as a 2‐ and 3‐year‐old. A control group of Standardbred racehorses that were contemporaneous paternal siblings of the affected horses was selected for comparison of all racing data. These horses were confirmed to be free of any OCD lesions using fluoroscopy, which is the routine screening method in the field for this study population.

### 2.4 Postoperative survey

Follow‐up for the operated horses was performed as an over‐the‐phone interview with owners or trainers, at least 1 year after surgery. The survey included questions regarding horse performance, persistence of lameness or joint effusion if present preoperatively, any postoperative complications, and overall satisfaction. The full owner survey is presented in Supplementary Material S1.

### 2.5 Statistical analysis

Statistical analyses were performed in the R computing environment.^13^ Normality was assessed using a Shapiro–Wilk test. Descriptive performance data are reported as median (interquartile range [IQR] and [range]). Interrater agreement for fragment measurements was assessed using intra‐class correlation coefficient (ICC). Multiple regression analysis was performed for all performance outcome variables. When evaluating the performance outcomes for the affected study cohort, the largest average size of the OCD lesion was the primary predictor variable of interest. For this analysis, whether the femoropatellar OCD lesions were bilateral (yes/no) or whether there were OCD lesions of other joints (yes/no) were included as covariates. When evaluating the performance outcomes for the entire study cohort (affected and matched controls), the OCD lesion was considered a dichotomous variable, and the presence or absence of OCD lesions was the primary predictor variable of interest. Sex and gait were included as covariates for all analyses as these are known to affect performance outcomes in Standardbred racehorses. Association with performance parameters was assessed with either linear regression (for continuous outcomes) or negative binominal regression (for count outcomes) in the affected individuals or affected and matched controls. Earnings ($) were log‐transformed for analyses. Additional predictor variables were added to the models as appropriate (e.g. number of starts as 2‐year‐old for the outcome variable wins as 2‐year‐old). Proportions (i.e. proportion of pacers and trotters) between affected and matched controls were compared using a two‐sample test for equality of proportions with continuity correction. Performance outcome variables of the affected cohort were also compared between lesion grades using a Wilcoxon signed‐rank test. Statistical significance was set at a *p*‐value <.05 for all analyses.

## 3 RESULTS

### 3.1 Study cohort

A total of 45 Standardbred racehorses were presented for arthroscopic removal of OCD lesions from the femoropatellar joint during the study period. A total of 32 horses had unilateral OCD lesions (32/45; 71%) and 13 horses had bilateral OCD lesions (13/45; 29%). Of the unilateral OCD lesions, the left femoropatellar joint was involved in 18 cases (56%) and the right femoropatellar joint was involved in the remaining 14 cases (44%). The median age was 1.68 years (IQR 1.50, 1.86, range: 0.83–2.45). None of the horses in the cohort had raced prior to surgery. This population included 17 fillies and 28 males (17 geldings and 11 colts). A total of 30 of the Standardbred racehorses were trotters (67%) and 15 were pacers (33%).

### 3.2 Lesions

There were unilateral OCD lesions of the lateral trochlear ridge of the femur (LTRF) in 25/45 horses (56%) and in 10/45 horses (22%) these lesions were observed bilaterally as shown in Table 1. A total of 10 horses were diagnosed with complex stifle pathology (presence of loose bodies or patella involvement); unilateral LTRF and loose bodies (4/45; 9%), unilateral LTRF and patella OCD (2/45; 4%), bilateral LTRF and loose bodies (2/45; 4%), and one horse (2%) with bilateral LTRF and patella OCD. There was one horse (2%) with a unilateral OCD lesion of the medial trochlear ridge of the femur. Some horses had mild cartilage irregularities noted intraoperatively; however, this was not extensive enough to impact their athletic potential as previously reported for the tibiotarsal joint.^2^

**TABLE 1:** Distribution of horses according to the location of the lesion (*n* = 45).

Location	Number of cases
Unilateral LTRF	25 (56%)
Bilateral LTRF	10 (22%)
Unilateral LTRF + loose bodies	4 (9%)
Unilateral LTRF + patella	2 (4%)
Bilateral LTRF + loose bodies	2 (4%)
Bilateral LTRF + patella	1 (2%)
Unilateral MTRF	1 (2%)

Abbreviations: LTRF, lateral trochlear ridge of the femur; MTRF, medial trochlear ridge of the femur.

All horses included in the study were screened for OCD lesions, and in addition to OCD lesions of the femoropatellar joint(s), 19 horses also had OCD lesions in other joints removed. Eight cases had an OCD lesion in the metacarpophalangeal or metatarsophalangeal joint (42.1%), five had an OCD lesion in the tibiotarsal joint (26.3%), three had an OCD lesion in both tibiotarsal joints (15.8%), and two had OCD lesions in both metatarsophalangeal joints (10.5%). One horse had OCD lesions in one tibiotarsal joint and one metatarsophalangeal joint (5.3%). None of the horses in this study presented with a history of lameness.

### 3.3 Radiographic measurements and grading

**FIGURE 1:**
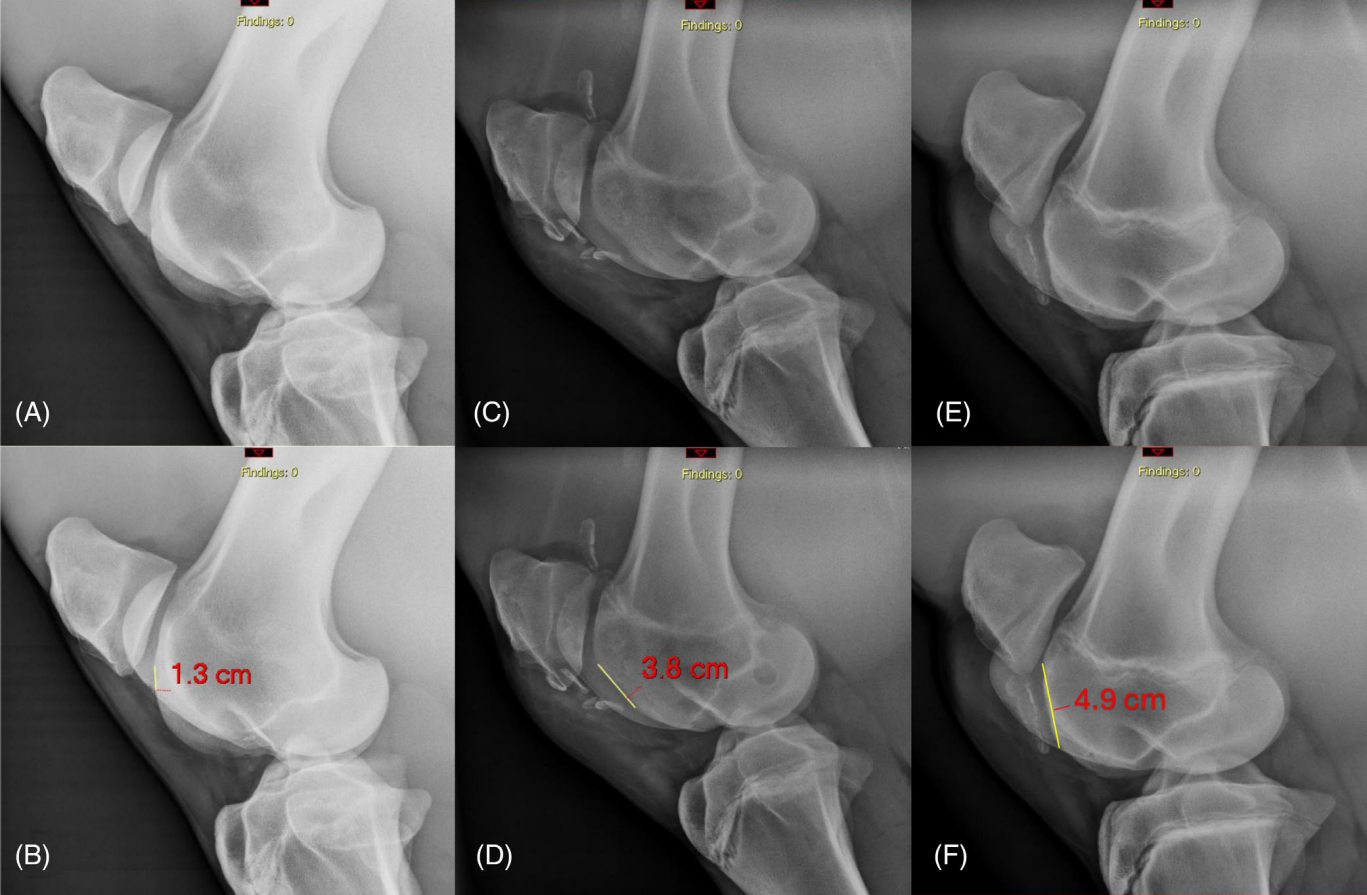
Representative latero‐medial radiographic projections of stifle joints diagnosed with osteochondrosis dissecans (OCD) lesions at the lateral trochlear ridge of the femur. (A, B) Grade 1, (C, D) grade 2 and (E, F) grade 3. (C, D) Also shows an example of loose bodies.

ICC for raters was 0.93 (95% CI: 0.88–0.96), indicating excellent agreement for lesion measurements. The median OCD lesion measurement was 2.4 cm (IQR 1.8 cm, 3.1 cm, [range: 0.9–4.9 cm]). There were 13 grade 1 OCD lesions (29%), 30 grade 2 OCD lesions (67%), and two grade 3 OCD lesions (4%). Radiographic examples of clinical cases for each grade are shown in Figure 1. Of the 13 horses that underwent bilateral femoropatellar arthroscopy, nine horses had bilateral lesions that were the same grade (1 horse with bilateral grade 1 lesions and 8 horses with bilateral grade 2 lesions). The horses with bilateral lesions that were of different grades were either grade 1 and grade 2 or grade 2 and grade 3. There were no horses with one joint that had a grade 1 OCD lesion, and the other joint with a grade 3 OCD lesion. For all analyses, the two horses with grade 3 lesions were included in the grade 2 group.

There was no difference in sex or gait distribution between horses with grade 1 OCD lesions and horses with grade 2 OCD lesions (*p* = 1). Of the 13 horses with grade 1 OCD lesions, eight were males (62%) and five were females (38%). For the 32 horses with grade 2 OCD lesions, 20 were males (63%) and 12 were females (37%). Of the 13 horses with grade 1 OCD lesions, four were pacers (31%) and nine were trotters (69%). For the 32 horses with grade 2 OCD lesions, 11 were pacers (34%) and 21 were trotters (66%).

### 3.4 Control cohort

A control group of 196 contemporaneous paternal Standardbred siblings were identified that were free of any OCD lesions (screened via fluoroscopy). This resulted in an approximately 4:1 ratio of unaffected: affected horses. This cohort included 95 fillies, 74 geldings, and 27 colts. There were 68 pacers and 128 trotters. There was no difference in sex distribution between affected and matched controls, with 28/45 (62%) males in the affected group and 101/196 (52%) males in the control group (*p* = .26). There was no difference in gait distribution between affected and matched controls, with 15/45 (33%) pacers and 30/45 (67%) trotters in the affected group and 68/196 (35%) pacers and 128/196 (65%) trotters in the control group (*p* = 1).

### 3.5 Racing performance prior

Racing data were available for 33 of the 45 Standardbreds (73%) included in the study cohort, while 12 horses had never raced. For the control group, racing data were available for 121 of the 196 horses (62%) and 75 of the horses never raced. Postoperative performance outcomes for the affected horses are summarized in Table 2. There was no difference in any of the performance outcomes evaluated between horses with grade 1 OCD lesions and horses with grade 2 OCD lesions.

**TABLE 2:** Summary of performance measures of affected horses in the study cohort. Data are reported as median (IQR) and [range]. *p*‐value reflects proportion test for “started a race” and Wilcoxon signed‐rank test for all other variables.

	Grade 1 (*n* = 13)	Grade 2 (*n* = 32)	*p*‐value
Started a race	10	23	1
	(77%)	(72%)	
Starts at 2	2 (0–8)	3.5 (0–7.75)	.90
	[0–18]	[0–15]	
Starts at 3	0 (0–18)	0 (0–15.75)	.85
	[0–27]	[0–27]	
Wins at 2	0 (0–1)	0 (0–0.25)	.42
	[0–10]	[0–11]	
Wins at 3	0 (0–3)	0 (0–2)	.60
	[0–8]	[0–7]	
Earnings at 2	$250 ($0–$9943)	$949 ($0–$4226)	.47
	[$0–$76 578]	[$0–196 750]	
Earnings at 3	$0 ($0–$12 410)	$0 ($0–$16 107)	.89
	[$0–$114 950]	[$0–$98 662]	
Fastest time (s)	117.1 (115.3–120.2)	118.2 (115.8–122.0)	.54
	[109.0–128.6] (*n* = 10)	[111.0–132.0] (*n* = 23)	

Abbreviation: IQR, interquartile range.

In the affected cohort (*n* = 45), performance outcomes were evaluated using the largest average size of the OCD lesion as the primary predictor variable of interest (Table S2). Most significant associations with performance in regression analysis were found in the 3‐year‐old season. Larger average lesion size was associated with fewer starts at 3 years of age (IRR = 0.84 [95% CI 0.74–0.96], *p* = .012), but the difference at 2 years of age, while of similar magnitude, did not reach statistical significance (IRR = 0.86 [95% CI: 0.72–1.03], *p* = .10). Females had fewer starts at 2 years of age (IRR = 0.71 [95% CI: 0.52–0.96], *p* = .03) and at 3 years of age (IRR = .37 [95% CI: 0.28–0.48], *p* < .0001) compared to males. As expected, trotters were slower than pacers (estimate 6.41 [95% CI: 2.56–10.25], *p* = .003). Horses with bilateral OCD lesions of the femoropatellar joint had more starts at 3 years of age than those with unilateral lesions (IRR = 1.41 [95% CI: 1.11–1.78], *p* = .005) but fewer wins (IRR = 0.38 [95% CI: 0.15–0.91], *p* = .03) and less earnings (estimate −0.61 [95% CI: −1.15 to −0.08], *p* = .03). Horses with OCD lesions in other joints had fewer starts at 3 years of age (IRR = 0.60 [95% CI: 0.47–0.76], *p* < .0001) than horses with femoropatellar joint OCD lesions only, but this was not associated with wins or earnings. The number of starts was a strong predictor of the number of wins and earnings for each race year.

For the entire study cohort (affected and matched controls), the presence or absence of an OCD lesion within the femoropatellar joint was the primary predictor variable of interest (Table S3). The presence of OCD lesions within the femoropatellar joint was associated with fewer starts at 3 years of age (IRR = 0.82 [95% CI: 0.73–0.91], *p* = .0004) and a slower qualifying race time (estimate 2.18 [95% CI: 0.47–3.89], *p* = .01). Females had fewer starts than males at both 2 and 3 years of age (2 years: IRR = 0.84 [95% CI: 0.75–0.94], *p* = .003; 3 years: IRR = 0.80 [95% CI: 0.73–0.87], *p* < .0001). Gait was associated with selected performance parameters. Trotters had fewer starts at 3 years of age than pacers (IRR = 0.77 [95% CI: 0.71–0.84], *p* < .0001) and were slower (estimate 4.19 [95% CI: 2.75–5.63], *p* < .0001). As in the affected cohort, the number of starts was strongly associated with the number of wins and earnings for each race year.

### 3.6 Follow‐up survey

Survey results were obtained via phone calls with owners or trainers for 34 of the 45 horses (76%) that underwent arthroscopic surgery of the femoropatellar joint(s). Median follow‐up time was 4 years (IQR 2 years, 5 years). There were no postoperative complications reported and all owners that responded to the survey, answered that they were very satisfied with the surgery.

## 4 DISCUSSION

This is the first study evaluating postoperative racing performance in a population of Standardbred racehorses following arthroscopic removal of OCD lesions from the femoropatellar joint. This study accounted for OCD lesion size and grade, as well as additional factors that could help predict postoperative racing performance. In this cohort of Standardbred racehorses, OCD lesions within the femoropatellar joint that were removed arthroscopically around one and 2 years of age, prior to the start of intensive race training had limited impact on postoperative racing performance. None of the 2‐year‐old racing outcomes were affected by OCD status. However, affected horses were predicted to have fewer starts at 3 years of age and slower qualifying race times when compared with unaffected siblings, accounting for other important factors such as sex and gait. Among the affected individuals, the size of the OCD lesion was only significantly associated with fewer starts at 3 years of age, suggesting that a good outcome can still be achieved even when the lesion is large. This was contrary to our expectations, as was the finding that bilateral lesions and the presence of OCD lesions in other joints each had a modest impact on racing performance postoperatively. In light of these results, our hypothesis was rejected.

Our findings compare favorably to previous studies focused on outcomes in Thoroughbreds. The proportion of affected Standardbred racehorses that started at least one race postoperatively (73%) was similar to both our unaffected control group (62%) and previous reports on Thoroughbreds (64%).^6^ Kerbert et al. also noted that affected Thoroughbreds and matched controls had no significant difference in the percentage of horses that started racing at 2 years of age.^11^ Sloan et al. showed affected Thoroughbreds had significantly fewer total starts, 3‐year‐old starts, 4‐year‐old starts, and 4‐year‐old places as compared to sibling controls.^14^ Although the authors concluded that the overall difference in these performance parameters was low.^14^ One previous study in Standardbred racehorses found no impact in short‐term racing performance; however, the longevity of their racing career was negatively impacted in affected horses as compared to non‐affected horses.^15^ It is widely accepted that not every horse bred for racing will make it to the track due to a variety of factors, including injury, illness, and lack of talent. However, an interesting finding in our cohort was that OCD grade did not significantly affect the proportion of starters (grade 1 and grade 2 were 77% and 72%, respectively). This contrasted with a previous study that showed horses with a grade 1 OCD lesion were more likely to race postoperatively (78%) compared to horses with a grade 2 OCD lesion (63%) or a grade 3 OCD lesion (54%).^6^ Clarke et al. previously found that horses with an OCD lesion grade of “moderate” or “severe” had significantly fewer wins at 3 years of age compared to horses with a “mild” OCD lesion grade.^10^ In their study, the grade of the OCD lesion was not significantly associated with any other racing outcomes.^10^ In both of these previous studies, the study population was mostly Thoroughbred racehorses.^6^, ^10^

Bilateral occurrence of OCD, or OCD affecting different joints at the same time, is common. In our cohort, 29% of horses (13/45) had bilateral lesions while 42% (19/45) had additional lesions in other joints. Both conditions had a limited impact on early racing performance. Horses with bilateral lesions had more starts at 3 years of age than those with unilateral lesions, but fewer wins and less earnings. Foland et al. reported a trend for horses with unilateral OCD lesions of the femoropatellar joint to be more likely to race postoperatively (67%) than horses with bilateral OCD lesions (61%),^6^ but the effect on wins and earnings was not reported. In our cohort, horses with OCD lesions removed from multiple joints had fewer starts at 3 years of age than those with femoropatellar OCD alone, but no other racing outcome was affected. In the previous study reporting performance outcome in Thoroughbreds,^6^ horses with OCD in multiple joints were less likely to successfully go on to race than those with lesions in a single joint, but more specific parameters of racing performance were not reported.

Additional factors that were found to affect early racing performance were sex and gait. Females had fewer starts at both 2 and 3 years of age. This is not surprising as mares can be moved to a breeding program instead of racing. However, geldings do not have the same option and are more likely to continue racing even if their results are not ideal. Within the affected cohort, gait did not impact the number of starts, but in the whole cohort, trotters started fewer races at 3 years of age. This may be because there are fewer opportunities for trotters to race compared to pacers, or it may reflect other factors not accounted for in this study. The finding that trotters were slower than pacers was also expected.^16^ Interestingly, even though wins and earnings were not associated with OCD status in our cohort, affected horses were slower, even when accounting for gait. This finding may be worth further study, especially since, within the affected group, there was no difference in fastest time between OCD lesion grades, and the largest lesion size was not associated with speed. It is possible that our study was underpowered to detect these associations; validation in a larger group of affected horses would be required.

This study had several limitations. As previously mentioned, the small affected sample size may have limited our power to detect true differences, especially between the different grades of OCD lesions. We only had two horses that fell within the most severe grade described by Foland et al.^6^ which led us to collapse our cases into only two categories. We addressed this limitation by evaluating lesion size as a continuous and categorical variable, but the impact of severe OCD lesions could not be addressed with the current cohort. There was also no conservative treatment group included in this study. We examined only short‐term performance parameters (through the 3‐year‐old racing season). It is possible that the true impact of femoropatellar OCD lesions affects athletic longevity rather than poor performance in the first two racing seasons, but this possibility is beyond the scope of the current study. Further work to investigate if there is any difference in racing longevity between affected horses and controls is warranted. Based on the findings in this population, we conclude that arthroscopy is an effective treatment for OCD lesions of the femoropatellar joint in Standardbred racehorses, when performed prior to the start of intensive training, and that lesions treated in this manner can be expected to have no major impact on early racing potential.

## AUTHOR CONTRIBUTIONS

Rhodes AR, DVM, MS: Contributed to data acquisition, analysis and interpretation, manuscript preparation, approved the final version of the manuscript, and agreed to be accountable. McCoy AM, DVM, MS, PhD, DACVS (Large Animal): Contributed to conception and design, data acquisition, analysis and interpretation, manuscript preparation, approved the final version of the manuscript, and agreed to be accountable. Stewart MC, BVSc, PhD, FACVSc, DACVSMR‐EQ: Contributed to interpretation of results, manuscript preparation, approved the final version of the manuscript, and agreed to be accountable. Gutierrez‐Nibeyro SD, DVM, MS, DACVS (Large Animal), DACVSMR‐EQ: Contributed to conception and design, data acquisition, analysis and interpretation, manuscript preparation, approved the final version of the manuscript, and agreed to be accountable.

## FUNDING INFORMATION

There were no grants, or any other sources of funding used for this study.

## CONFLICT OF INTEREST STATEMENT

The authors declare no conflicts of interest.

## Supporting information


**Supplementary Material S1:** Owner survey used to perform follow‐up interviews. Owners were contacted by phone at least 1 year after surgery to obtain follow‐up.
**Table S2.** Regression analysis results for performance outcomes for affected study cohort using largest average lesion size as the predictor variable. F, female; IRR, incident rate ratio; M, male; P, pace; T, trot. The reference group for effect estimates is denoted by REF. *p*‐values in bold are significant.
**Table S3.** Regression analysis results for performance outcomes for entire study cohort. OCD lesions were considered as a dichotomous variable (present/absent). F, female; IRR, incident rate ratio; M, male; OCD, osteochondrosis dissecans; P, pace; T, trot. The reference group for effect estimates is denoted by REF. *p*‐values in bold are significant.
