# Supplementary material for: Outcome of Standardbred racehorses following femoropatellar arthroscopy for osteochondrosis dissecans
Source: Vet Surg. 2025 Nov 21;55(5):976–83. doi: 10.1111/vsu.70058 (PMC13360759; doi:10.1111/vsu.70058)
Supplement: Supplementary file 1 — Supplementary Material S1: Owner survey used to perform follow‐up interviews. Owners were contacted by phone at least 1 year after surgery to obtain follow‐up. Table S2. Regression analysis results for performance outcomes for affected study cohort using largest average lesion size as the predictor variable. F, female; IRR, incident rate ratio; M, male; P, pace; T, trot. The reference group for effect estimates is denoted by REF. p‐values in bold are significant. Table S3. Regression analysis results for performance outcomes for entire study cohort. OCD lesions were considered as a dichotomous variable (present/absent). F, female; IRR, incident rate ratio; M, male; OCD, osteochondrosis dissecans; P, pace; T, trot. The reference group for effect estimates is denoted by REF. p‐values in bold are significant. [file VSU-55-976-s001.docx]

**Supplementary Material S1:** Owner survey used to perform follow-up interviews. Owners were contacted by phone at least one year after surgery to obtain follow-up.

**We are following up on horses that have had stifle surgery at University of Illinois VTH since 2014.**

1. Was the horse in WORK or TRAINING before undergoing surgery? (circle one)

2. If yes, what was the horses’ use/intended use? _______________________________

3. Did the horse return to its previous level of use or go on to its intended use?

YES NO (circle one)

3a. If yes, how long until return to work? ________________

→ Is the horse still in work? YES NO If no, why not?____________________________

3b. If no, was the failure to return to work due to the stifle condition? YES NO (circle one)

→ What was the horse able to do after surgery (e.g. breeding, trail ride, other competition, pasture sound, etc.)? ___________________________________

4. Did the horse receive any additional therapy in the stifle(s)?

Steroids __________ Other____________________________________

HA/Adequan __________

IRAP __________

Stem Cells __________

5. Is (was) the horse on any medications or supplements to address joint health or comfort?

NSAIDs (Bute/Banamine/Equioxx) __________

Legend IV __________

Adequan IM __________

Glucosamine/Chondroitin __________

Other____________________________________________________________

**Supplementary Material S1 (cont’d)**

6. How satisfied with the surgery are you?

Very satisfied __________ Moderately satisfied___________

Neither satisfied nor dissatisfied__________

Moderately dissatisfied__________ Very dissatisfied___________

7. Were there any postoperative complications (e.g. incisional drainage, persistent effusion, persistent lameness, etc.)?

YES NO (circle one) If yes, specify _______________________

8. Do you have any other comments you would like to make? _____________________

**Supplementary Table S2:** Regression analysis results for performance outcomes for affected study cohort using largest average lesion size as the predictor variable. M = male; F = female; P = pace; T = trot; IRR = incident rate ratio. The reference group for effect estimates is denoted by REF. P-values in **bold** are significant.

| **Outcome Variable** | | **Predictor Variable** | | **Estimate (IRR)** | | **95% Confidence Interval** | | | | **p-value** | |
| --- | --- | --- | --- | --- | --- | --- | --- | --- | --- | --- | --- |
|  |  |  |  |  |  | **2.5%** | | **97.5%** | |  |  |
| Starts at 2 (number) | | Largest Avg | | 0.86 | | 0.72 | | 1.03 | | 0.10 | |
|  |  | Sex (M) | | REF | | n/a | | n/a | | n/a | |
|  |  | Sex (F) | | 0.71 | | 0.52 | | 0.96 | | **0.03** | |
|  |  | Gait (P) | | REF | | n/a | | n/a | | n/a | |
|  |  | Gait (T) | | 0.82 | | 0.62 | | 1.10 | | 0.18 | |
|  |  | Bilat (N) | | REF | | n/a | | n/a | | n/a | |
|  |  | Bilat (Y) | | 1.05 | | 0.75 | | 1.45 | | 0.77 | |
|  |  | Other Joints (N) | | REF | | n/a | | n/a | | n/a | |
|  |  | Other Joints (Y) | | 0.75 | | 0.55 | | 1.01 | | 0.06 | |
|  | | | | | | | | | | | |
| Starts at 3 (number) | | Largest Avg | | 0.84 | | 0.74 | | 0.96 | | **0.012** | |
|  |  | Sex (M) | | REF | | n/a | | n/a | | n/a | |
|  |  | Sex (F) | | 0.37 | | 0.28 | | 0.48 | | **<0.0001** | |
|  |  | Gait (P) | | REF | | n/a | | n/a | | n/a | |
|  |  | Gait (T) | | 0.83 | | 0.67 | | 1.03 | | 0.09 | |
|  |  | Bilat (N) | | REF | | n/a | | n/a | | n/a | |
|  |  | Bilat (Y) | | 1.41 | | 1.11 | | 1.78 | | **0.005** | |
|  |  | Other Joints (N) | | REF | | n/a | | n/a | | n/a | |
|  |  | Other Joints (Y) | | 0.60 | | 0.47 | | 0.76 | | **<0.0001** | |
|  | | | | | | | | | | | |
| Wins at 2 (number) | | Largest Avg | | 0.46 | | 0.15 | | 1.21 | | 0.12 | |
|  |  | Sex (M) | | REF | | n/a | | n/a | | n/a | |
|  |  | Sex (F) | | 1.94 | | 0.56 | | 7.17 | | 0.31 | |
|  |  | Gait (P) | | REF | | n/a | | n/a | | n/a | |
|  |  | Gait (T) | | 1.16 | | 0.38 | | 3.67 | | 0.79 | |
|  |  | Starts at 2 | | 1.42 | | 1.26 | | 1.68 | | **<0.0001** | |
|  |  | Bilat (N) | | REF | | n/a | | n/a | | n/a | |
|  |  | Bilat (Y) | | 1.24 | | 0.29 | | 5.90 | | 0.78 | |
|  |  | Other Joints (N) | | REF | | n/a | | n/a | | n/a | |
|  |  | Other Joints (Y) | | 1.25 | | 0.32 | | 4.88 | | 0.73 | |
|  | | | | | | | | | | | |
| Wins at 3 (number) | | Largest Avg | | 0.85 | | 0.56 | | 1.25 | | 0.41 | |
|  |  | Sex (M) | | REF | | n/a | | n/a | | n/a | |
|  |  | Sex (F) | | 0.63 | | 0.25 | | 1.49 | | 0.31 | |
|  |  | Gait (P) | | REF | | n/a | | n/a | | n/a | |
|  |  | Gait (T) | | 0.92 | | 0.47 | | 1.82 | | 0.80 | |
|  |  | Starts at 3 | | 1.17 | | 1.11 | | 1.24 | | **<0.0001** | |
|  |  | Bilat (N) | | REF | | n/a | | n/a | | n/a | |
|  |  | Bilat (Y) | | 0.38 | | 0.15 | | 0.91 | | **0.03** | |
|  |  | Other Joints (N) | | REF | | n/a | | n/a | | n/a | |
|  |  | Other Joints (Y) | | 0.55 | | 0.24 | | 1.21 | | 0.13 | |
|  | | | | | | | | | | | |
| **Outcome Variable** | **Predictor Variable** | | **Estimate** | | **95% Confidence Interval** | | | | **p-value** | |  |
|  |  |  |  |  | **2.5%** | | **97.5%** | |  |  |  |
| Earnings 2 (log[$]) | Largest Avg | | 0.20 | | -0.15 | | 0.84 | | 0.27 | |  |
|  | Sex (M) | | REF | | n/a | | n/a | | n/a | |  |
|  | Sex (F) | | 0.43 | | -0.16 | | 1.01 | | 0.16 | |  |
|  | Gait (P) | | REF | | n/a | | n/a | | n/a | |  |
|  | Gait (T) | | 0.06 | | -0.53 | | 0.66 | | 0.83 | |  |
|  | Starts at 2 | | 0.33 | | 0.28 | | 0.38 | | **<0.0001** | |  |
|  | Bilat (N) | | REF | | n/a | | n/a | | n/a | |  |
|  | Bilat (Y) | | -0.18 | | -0.83 | | 0.48 | | 0.60 | |  |
|  | Other Joints (N) | | REF | | n/a | | n/a | | n/a | |  |
|  | Other Joints (Y) | | 0.21 | | -0.39 | | 0.81 | | 0.49 | |  |
|  | | | | | | | | | | |  |
| Earnings 3 (log[$]) | Largest Avg | | 0.03 | | -0.26 | | 0.31 | | 0.85 | |  |
|  | Sex (M) | | REF | | n/a | | n/a | | n/a | |  |
|  | Sex (F) | | -0.28 | | -0.77 | | 0.22 | | 0.28 | |  |
|  | Gait (P) | | REF | | n/a | | n/a | | n/a | |  |
|  | Gait (T) | | -0.39 | | -0.87 | | 0.10 | | 0.13 | |  |
|  | Starts at 3 | | 0.21 | | 0.19 | | 0.24 | | **<0.0001** | |  |
|  | Bilat (N) | | REF | | n/a | | n/a | | n/a | |  |
|  | Bilat (Y) | | -0.61 | | -1.15 | | -0.08 | | **0.03** | |  |
|  | Other Joints (N) | | REF | | n/a | | n/a | | n/a | |  |
|  | Other Joints (Y) | | -0.16 | | -0.65 | | 0.32 | | 0.51 | |  |
|  | | | | | | | | | | |  |
| Fastest Time (secs) | Largest Avg | | 0.13 | | -2.02 | | 2.28 | | 0.91 | |  |
|  | Sex (M) | | REF | | n/a | | n/a | | n/a | |  |
|  | Sex (F) | | 3.73 | | 0.09 | | 7.37 | | 0.05 | |  |
|  | Gait (P) | | REF | | n/a | | n/a | | n/a | |  |
|  | Gait (T) | | 6.41 | | 2.56 | | 10.25 | | **0.003** | |  |
|  | Bilat (N) | | REF | | n/a | | n/a | | n/a | |  |
|  | Bilat (Y) | | 1.88 | | -2.33 | | 6.09 | | 0.39 | |  |
|  | Other Joints (N) | | REF | | n/a | | n/a | | n/a | |  |
|  | Other Joints (Y) | | 2.31 | | -1.93 | | 6.55 | | 0.30 | |  |

**Supplementary Table S3:** Regression analysis results for performance outcomes for entire study cohort. OCD lesions were considered as a dichotomous variable (present/absent). OCD = osteochondrosis dissecans; M = male; F = female; P = pace; T = trot; IRR = incident rate ratio. The reference group for effect estimates is denoted by REF. P-values in **bold** are significant.

| **Outcome Variable** | **Predictor Variable** | **Estimate (IRR)** | **95% Confidence Interval** | | **p-value** |
| --- | --- | --- | --- | --- | --- |
|  |  |  | **2.5%** | **97.5%** |  |
| Starts at 2 (number) | OCD (no) | REF | n/a | n/a | n/a |
|  | OCD (yes) | 0.93 | 0.80 | 1.07 | 0.33 |
|  | Sex (M) | REF | n/a | n/a | n/a |
|  | Sex (F) | 0.84 | 0.75 | 0.94 | **0.003** |
|  | Gait (P) | REF | n/a | n/a | n/a |
|  | Gait (T) | 0.90 | 0.80 | 1.01 | 0.07 |
|  | | | | | |
| Starts at 3 (number) | OCD (no) | REF | n/a | n/a | n/a |
|  | OCD (yes) | 0.82 | 0.73 | 0.91 | **0.0004** |
|  | Sex (M) | REF | n/a | n/a | n/a |
|  | Sex (F) | 0.80 | 0.73 | 0.87 | **<0.0001** |
|  | Gait (P) | REF | n/a | n/a | n/a |
|  | Gait (T) | 0.77 | 0.71 | 0.84 | **<0.0001** |
|  | | | | | |
| Wins at 2 (number) | OCD (no) | REF | n/a | n/a | n/a |
|  | OCD (yes) | 0.90 | 0.54 | 1.50 | 0.69 |
|  | Sex (M) | REF | n/a | n/a | n/a |
|  | Sex (F) | 0.84 | 0.56 | 1.26 | 0.42 |
|  | Gait (P) | REF | n/a | n/a | n/a |
|  | Gait (T) | 1.40 | 0.92 | 2.14 | 0.12 |
|  | Starts at 2 | 1.34 | 1.28 | 1.40 | **<0.0001** |
|  | | | | | |
| Wins at 3 (number) | OCD (no) | REF | n/a | n/a | n/a |
|  | OCD (yes) | 1.0 | 0.63 | 1.59 | 0.99 |
|  | Sex (M) | REF | n/a | n/a | n/a |
|  | Sex (F) | 0.77 | 0.53 | 1.12 | 0.16 |
|  | Gait (P) | REF | n/a | n/a | n/a |
|  | Gait (T) | 1.24 | 0.85 | 1.80 | 0.26 |
|  | Starts at 3 | 1.15 | 1.13 | 1.18 | **<0.0001** |
|  | | | | | |
| **Outcome Variable** | **Predictor Variable** | **Estimate** | **95% Confidence Interval** | | **p-value** |
|  |  |  | **2.5%** | **97.5%** |  |
| Earnings at 2 (log[$]) | OCD (no) | REF | n/a | n/a | n/a |
|  | OCD (yes) | -0.03 | -0.32 | 0.27 | 0.86 |
|  | Sex (M) | REF | n/a | n/a | n/a |
|  | Sex (F) | 0.02 | -0.22 | 0.26 | 0.87 |
|  | Gait (P) | REF | n/a | n/a | n/a |
|  | Gait (T) | 0.17 | -0.08 | 0.42 | 0.18 |
|  | Starts at 2 | 0.33 | 0.31 | 0.35 | **<0.0001** |
|  | | | | | |
| Earnings at 3 (log[$]) | OCD (no) | REF | n/a | n/a | n/a |
|  | OCD (yes) | -0.19 | -0.51 | 0.13 | 0.24 |
|  | Sex (M) | REF | n/a | n/a | n/a |
|  | Sex (F) | -0.10 | -0.35 | 0.15 | 0.42 |
|  | Gait (P) | REF | n/a | n/a | n/a |
|  | Gait (T) | 0.14 | -0.13 | 0.40 | 0.31 |
|  | Starts at 3 | 0.19 | 0.18 | 0.20 | **<0.0001** |
|  | | | | | |
| Fastest Time (secs) | OCD (no) | REF | n/a | n/a | n/a |
|  | OCD (yes) | 2.18 | 0.47 | 3.89 | **0.01** |
|  | Sex (M) | REF | n/a | n/a | n/a |
|  | Sex (F) | 0.85 | -0.56 | 2.26 | 0.24 |
|  | Gait (P) | REF | n/a | n/a | n/a |
|  | Gait (T) | 4.19 | 2.75 | 5.63 | **<0.0001** |
